# Supplementary material for: Long non-coding RNA Cerox1 targets components of the mitochondrial electron transport chain to regulate the memory impairment caused by sleep deprivation
Source: Res Sq. 2025 Sep 29:rs.3.rs-7518376. Preprint. [Version 1] doi: 10.21203/rs.3.rs-7518376/v1 (PMC12622179; doi:10.21203/rs.3.rs-7518376/v1)
Supplement: Supplement 1 [file NIHPPRS7518376V1-supplement-1.pdf]

Supplementary Data

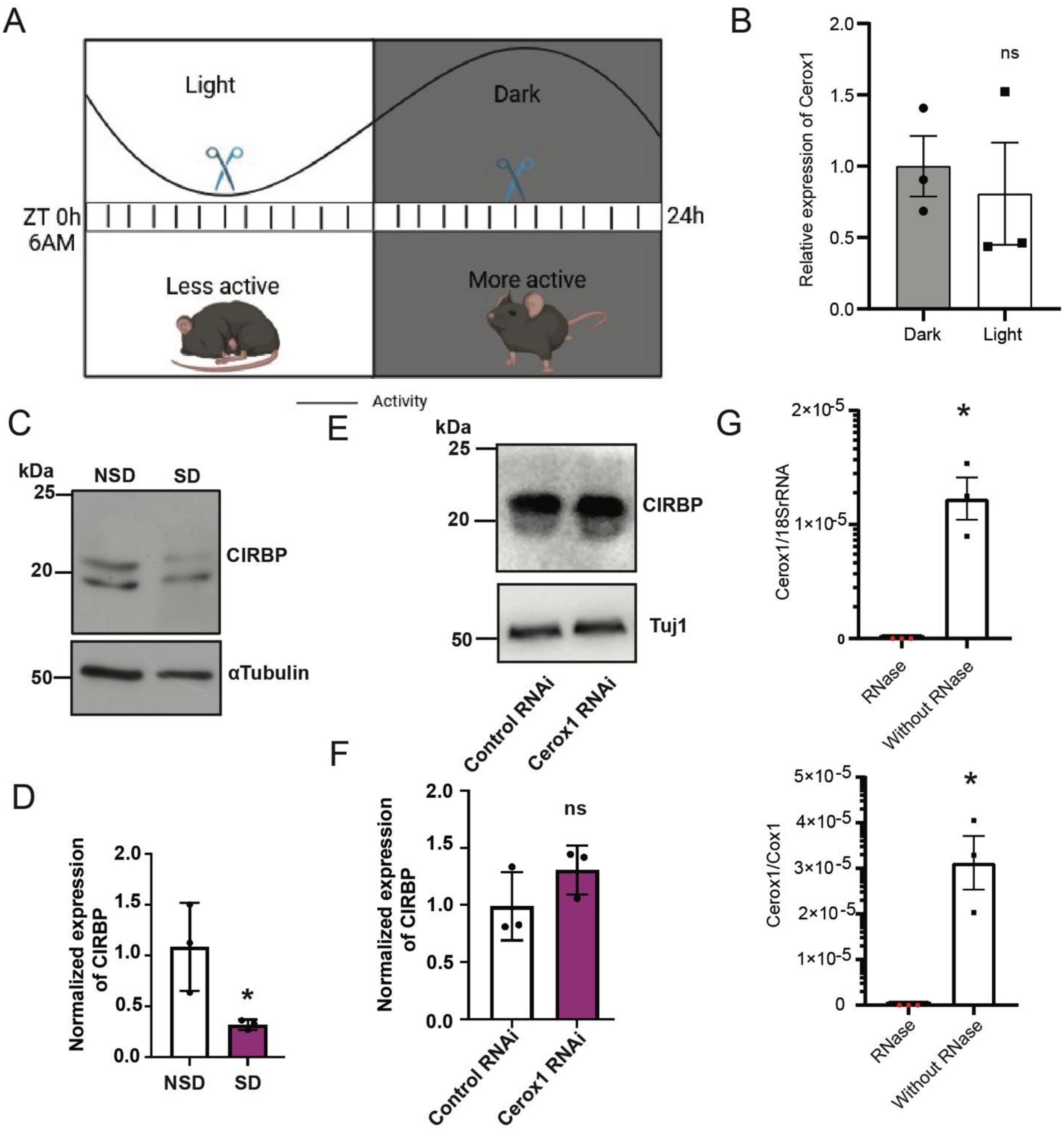

Figure S1: Abundance of Cerox1 during circadian cycle and in mitochondria

(A). Schematic representation of circadian cycle showing sleep and awake cycle. Hippocampus was harvested as indicated during the cycle. **(B).** qPCR analysis of Cerox1 abundance in Dark (awake) and Light (sleep) phase of circadian cycle. **(C).** Western blot showing expression of CIRBP from NSD or SD mice. **(D).** Quantification of western blot.

n = 3, *p = 0.0383, unpaired two tailed *t-test*. **(E).** Western blot showing CIRBP expression in presence or absence of Cerox1. **(F).** Quantification of western blot. n = 3, unpaired two tailed *t-test*. ns = not significant. **(G).** Abundance of Cerox1 in mitochondrial fraction treated with or without RNase. qPCR analysis showing Cerox1 expression as normalized by 18S rRNA and mitochondria specific Cox1 transcript. n = 3, Data represents mean ± SEM. “n” represents biological replicates. Data represents mean ± SEM. “n” represents biological replicates.

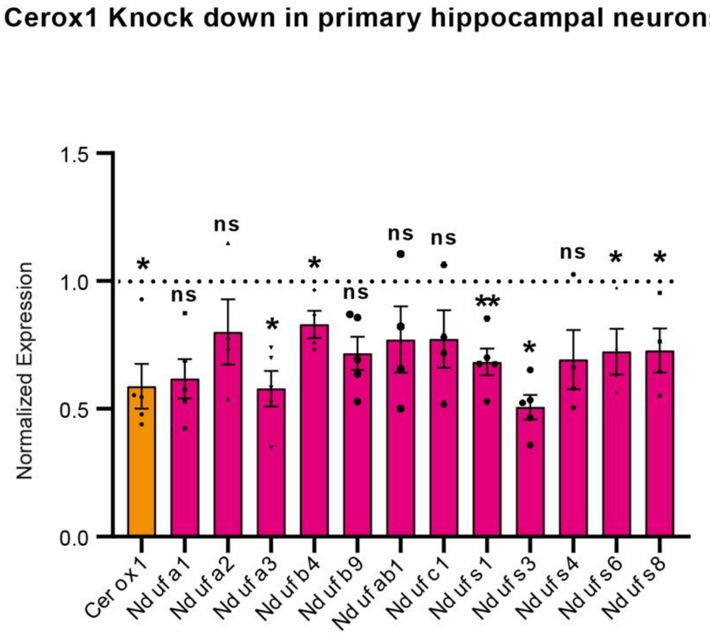

Figure S2: Expression of complex I subunits following Cerox1 knockdown in primary hippocampal neurons.

qPCR profiling of 12 complex I subunits and Cerox1 from the dorsal hippocampus expressing Cerox1 or control shRNA. Cerox1, n=5, *p=0.047; Ndufa1, n=5, p =0.056; Ndufa2, n=4, *p=0.219; Ndufa3, n=5, *p=0.026; Ndufb4, n=4, *p=0.042; Ndufb9, n=5, p=0.07; Ndufab1, n=4, p=0.174; Ndufc1, n=4, p=0.088; Ndufs1, n=5, **p=0.0016; Ndufs3, n=5, *p=0.034; Ndufs4, n=4, p=0.07; Ndufs6, n=4, *p=0.046; Ndufs8, n=4, *p =0.032. Statistical analysis between groups performed using two-tailed t-test with Welch’s correction. Data represents mean ± SEM. “n” represents biological replicates.
